# The link between T cell activation and development of functionally useful tumour-associated high endothelial venules

**DOI:** 10.1093/discim/kyad006

**Published:** 2023-04-24

**Authors:** Stefan Milutinovic, Awen Gallimore

**Affiliations:** Systems Immunity University Research Institute, Henry Wellcome Building, School of Medicine, Cardiff University, Cardiff, UK; Systems Immunity University Research Institute, Henry Wellcome Building, School of Medicine, Cardiff University, Cardiff, UK

**Keywords:** high endothelial venules, T-cell activation, immune checkpoint blockade, lymphotoxin-β receptor agonist

## Abstract

High endothelial venules (HEVs) are specialized postcapillary venules that specifically serve to recruit circulating lymphocytes to secondary lymphoid organs (SLOs) where cognate antigens can be encountered, and immune responses can be initiated. The presence of HEV-like vessels in primary human solid tumours and their association with lymphocyte infiltration and favourable clinical outcomes and response to immunotherapy have provided a rationale for therapeutically inducing these vessels in tumours for immunotherapeutic benefit. Here we specifically discuss evidence for a link between T-cell activation and development of useful tumour-associated HEV (TA-HEV). We discuss the molecular and functional features of TA-HEV, highlighting the benefits for promoting tumour immunity and the important unanswered questions that need to be addressed before TA-HEV induction can be optimized for immunotherapeutic benefit.

## Graphical Abstract

**Graphical Abstract:**
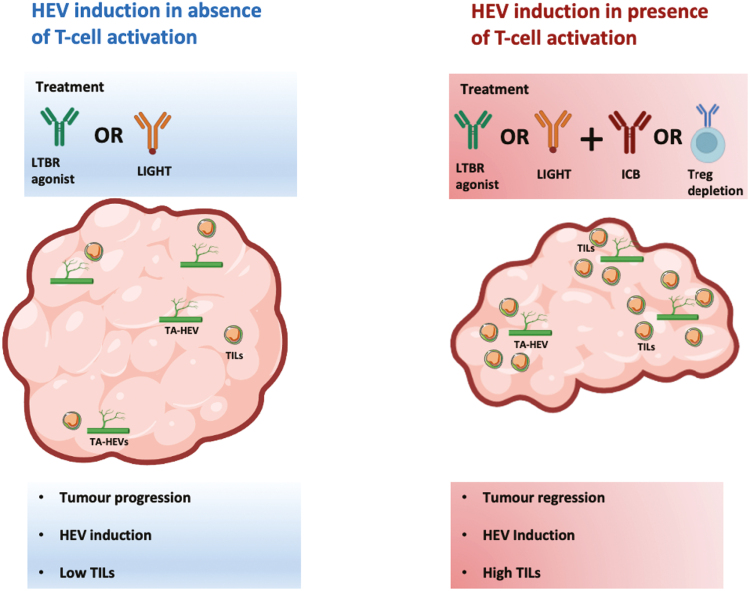

## HEVs in human cancer

HEVs have been described in both human cancer and chronic inflammatory diseases where they can be found in isolation or as parts of lymphoid-like tissue, termed tertiary lymphoid structures (TLS). TLS can vary in their respective organizational capacity but are not encapsulated like LNs [1]. The role of HEVs in chronic inflammatory conditions [2] and TLS in cancer have been extensively reviewed elsewhere [3, 4]. The presence of TA-HEVs in primary human solid tumours such as melanomas, lung, and breast carcinomas was first described by Martinet *et al*. [5]. TA-HEVs were found to be associated with lymphocyte infiltration which was further linked to favourable clinical outcome [5–8]. Similar findings have since been reported in several other tumour types [9–11]. More recently however, the presence of TA-HEVs has also been implicated in favourable responses to immunotherapy [12, 13]. Pre-treatment lesions with high TA-HEV scores from patients with unresectable stage III or IV metastatic melanoma treated with PD-1 blockade therapy had improved survival and treatment responses to combined immune checkpoint blockade (ICB) [12]. Furthermore, a human HEV gene signature was also shown to be associated with better responses to ICB in pre-treated samples obtained from melanoma (treated with nivolumab) and non-small cell lung cancer (NSCLC) patients (treated with atezolizumab) [13]. Melanoma patients with stable disease were also shown to have a significant increase in TA-HEV score following ICB [13]. Therefore, the presence of TA-HEVs in human cancer has important implications for both clinical outcome and response to immunotherapy.

## Spontaneous HEV formation in mouse models of cancer

Spontaneous formation of TA-HEVs has largely been observed in murine tumours which express strong antigens such as ovalbumin (OVA) [14] or large-T antigen of simian virus 40 (SV40 Tag) [15]. However, TA-HEV formation has also been observed in widely used tumour cell lines such as the 4T1 murine mammary tumour model [16]. Differences in the type of tumour models (cell line-derived vs *in-situ*) and site of tumour implantation [intraperitoneal (i.p.) vs subcutaneous (s.c.)] may point to important factors which regulate HEV formation and function. HEVs are readily detected by staining with the MECA-79 antibody which recognizes the 6-sulpho sialyl Lewis X epitope presented on peripheral node addressin (PNAd) [17]. PNAd is considered a marker of functional HEVs as it an adhesion molecule which binds CD62L (L-selectin) and mediates the tethering and rolling of lymphocytes along HEVs [17]. Peske and colleagues described spontaneously arising TA-HEVs in B16 and Lewis carcinoma cell lines engineered to express strong antigens such as OVA or a tyrosinase epitope [14]. Interestingly, B16-F1 tumours which lack a strong antigen were found to have significantly lower PNAd expression [14] suggesting immune activation as an important component in spontaneous TA-HEV formation. Furthermore, the immune cell subsets which regulate PNAd expression were found to differ depending upon the site of tumour implantation [14]. For instance, in s.c. tumours, NK cells were found to act redundantly with endogenous CD8 T cells to induce PNAd expression whilst in i.p. tumours, CD8 T cells were absolutely required for PNAd expression [14].

Indeed, in a mouse model of carcinogen-induced fibrosarcoma, the depletion of regulatory T cells (Tregs) was found to drive the development of TA-HEVs which were associated with reduced tumour growth and increased tumour infiltrating lymphocyte (TIL) frequencies [18, 19]. Immune activation via Treg depletion was a pre-requisite for TA-HEV formation since untreated fibrosarcoma tumours did not spontaneously form TA-HEVs. In contrast, cell lines derived from MCA-induced fibrosarcomas inoculated in the flank of syngeneic mice were found to spontaneously develop TA-HEVs [12]. This suggests that there may be important differences between tumours which arise from tumour cell line inoculation versus those which develop *in situ*. Factors such as tumour immunogenicity or underlying vascular differences may hold important clues to why TA-HEVs form in some tumours but not others. For example, tumours arising *in vivo* from injected cells lines are more angiogenic and comprise morphologically immature vessels that have been shown to be more sensitive to vascular disruption in response to inflammatory cytokines such as tumour necrosis factor (TNF) [20]. Nevertheless, it is apparent that immune activation is an important component in TA-HEV formation in both cell line-derived tumours and those which arise *in situ*. Immune activation can also enhance TA-HEV formation in cell line-derived tumours. For example, 4T1 tumour cells inoculated in the mammary fat pad of Balb/c mice were shown to spontaneously form TA-HEVs in approximately 18% of mice. Inactivation of Treg through targeting of the PI3Kδ pathway lead to the majority of treated mice (89%) forming TA-HEVs with higher TA-HEV areas reported in regressor tumours [16]. Recently, 3D imaging of whole 4T1 tumours revealed denser TA-HEV networks in regressors as compared to non-regressors and control, untreated tumours [21]. Overall, these data imply that tumour immunogenicity is linked to intra-tumoural HEV development. Indeed TA-HEVs have been shown to spontaneously form in several immunogenic human tumours including melanoma and NSCLC [5]. In poorly immunogenic tumours such as human colorectal cancer (CRC), TA-HEV were rarely observed within CRC tumour stroma or epithelium and were mainly localized to the tumour invasive margin [22]. Furthermore, microsatellite instability (MSI) CRC tumours which have a high load of mutational neoantigens due to DNA mismatch repair deficiencies were shown to have elevated TA-HEV densities as compared to microsatellite-stable tumours [23], further supporting the link between tumour immunogenicity and TA-HEV development.

## TA-HEVs as active sites for immune cell infiltration

What roles might TA-HEVs play in the context of anti-tumour immunity? As in SLOs, several studies have implicated TA-HEVs as active sites for immune cell recruitment. For example, intravenously injected GFP^+^ splenocytes can be recruited to spontaneously induced TLS containing TA-HEVs in a model of inflammation-driven carcinogenesis [24]. Furthermore, the induction of TLS in splenectomised LTα^–/–^ mice, which lack all peripheral LNs, leads to the recruitment and induction of specific T cell responses in a B16 tumour model, suggesting *in situ* priming at TLS sites [25]. In B16-OVA expressing tumours, the administration of blocking antibodies targeted against L-selectin (which recognizes sulfated sialomucins displayed on HEVs) or anti-CCR7 (activates LFA-1 leading to lymphocyte arrest) prevented lymphocyte infiltration into tumours [14]. Similarly, for fibrosarcomas induced by the administration of the carcinogen methylcholanthrene (MCA), blocking the TNFR signalling pathway which abrogates TA-HEV development resulted in a TIL frequency comparable to those tumours that do not develop TA-HEVs following Treg depletion [18]. Whilst several studies have provided evidence for an active role of TA-HEVs in driving immune cell recruitment, the presence of TA-HEVs may simply be a marker of an ongoing immune response given that factors released by immune cells can drive TA-HEV formation [1, 26]. With this in mind, it has proven difficult to uncouple the ability of immune cells to drive TA-HEV development from the ability of TA-HEV to enable recruitment of T cells into the tumour parenchyma.

**Figure 1:**
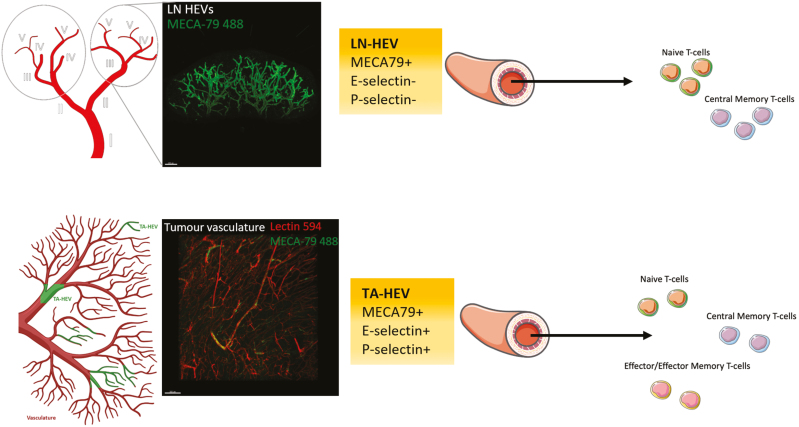
Branching structure and function of LN HEVs vs TA-HEVs. LN HEVs are embedded in a distinct hierarchy of venules branching out from the largest collecting vein (order I) to the smallest postcapillary venules (order V). LN-HEVs constitute order III-V venules and are major sites of naive T cell and central memory T cell recruitment. In contrast TA-HEVs do not form a distinct branching hierarchy and can be identified as part of the larger blood vascular network by intravascular staining using fluorescent MECA-79 mAbs (HEV marker) and lectin 594 (blood vessel marker). In addition to recruiting naïve T cells and central memory T cells which express L-selectin (CD62L^+^), TA-HEV express high levels of P-selectin and E-selectin and have been shown to recruit effector/effector memory T cells (CD62L^-^) from the periphery [17]. LN HEV network depicted is from a naïve inguinal LN labelled with 20 µg MECA-79 488 and imaged by selective plane illumination microscopy (SPIM) [21]. Tumour vasculature depicted is from a Treg depleted fibrosarcoma tumour labelled with 20 µg MECA-79 488 and 100 µg lectin 594 and imaged by SPIM. Figure adapted from [17, 26].

Only recently have TA-HEVs been shown to directly act as the main sites for immune cell recruitment in three separate tumour cell line models [12]. By inoculating mice with cell lines derived from MCA-induced fibrosarcomas in WT mice (termed MCAprog tumours), Asrir *et al*. demonstrated by intra-vital 2-photon imaging that TA-HEVs constitute the major sites of lymphocyte arrest in the microvasculature [12]. This was directly shown by comparing lymphocytes that transitioned from rolling to firm arrest in MECA-79- non-HEV tumour blood vessels to MECA-79+ HEV tumour blood vessels. Similar results were obtained for mice treated with ICB (combined CTLA4 and PD-1 blockade therapy) which further boosts TA-HEV formation [12]. This was also confirmed in two separate mouse models of ICB-treated CT26 colon carcinomas and PyMT mammary carcinomas [12]. Overall, this work is important as it expands upon early intravital microscopy studies which revealed that the major sites of lymphocyte recruitment in LNs occurs in venule branches that are of the order III–V (Fig. 1) [27]. Indeed, similarities and differences were noted between the two studies which may point to different functionalities. For example, the mean diameter of TA-HEVs (40 µm) was found to be similar to that of order III HEVs found in LNs [27] while higher median lymphocyte rolling velocities were noted in TA-HEVs as compared to LNs [12]. Indeed, such differences may be explained by underlying transcriptional differences which were revealed by singe-cell RNA sequencing (scRNA-seq) of spontaneously forming tumour-associated high endothelial cells (TA-HECs) [12] that were subsequently compared to homeostatic LN HECs and inflamed LN HECs (iLN-HECs) [28]. TA-HECs were found to be associated with an immature phenotype characterized by reduced expression of HEC sulfotransferases and lymphotoxin β receptor (LTβR) pathway dependant genes [12]. TA-HECs were also found to more closely resemble iLN-HECs with the shared expression of 250 genes which were otherwise differentially expressed with homeostatic LN-HECs [12]. However, TA-HECs were also found to uniquely express several genes that were either absent or poorly expressed in the LN setting [12]. For example, the endothelial selectins CD62P and CD62E, were found to be uniquely upregulated in TA-HECs. This may explain why TA-HECs were capable of recruiting both CD62L^−^ effector/effector memory T-cells and CD62L^+^ naïve and central memory T cells in short-term homing assays [12]. This discord between LN HEVs and TA-HECs was also recently demonstrated by scRNA-seq of TA-HECs isolated from PyMT-bearing mice treated with ICB (PD-L1 blockade), VEGFR2 blockade and LTβR agonist which induces TA-HEV formation [13]. TA-HECs were shown to express both core HEV markers (such as Fut7 and Glycam1) and genes found in TA-ECs (Gpihbp1, Igfbp3, and Cxcl9). However, TA-HECs were also shown to harbour key inflammatory markers (CD62P and CD62E) and to uniquely express IFN-gamma regulated inflammation genes such as those involved in antigen processing and presentation [13]. Interestingly, blocking IFN-y had no impact on T-cell influx or the extent of TA-HEV formation however TA-HECs were found to have an increased expression of core LN-HEV genes suggesting a reversion back to homeostatic LN-HECs [13]. Overall, these data indicate that the molecular and functional features of TA-HEV are similar but not identical to those of LN-HEV. These differences have important implications for understanding how TA-HEV promote tumour immunity.

## Therapeutic induction of HEV in murine tumours

### Targeting of the LTβR signalling pathway

The LTβR pathway has been implicated in early SLO development [29] and in the formation and maintenance of HEVs [5, 30–34]. Indeed, LTβR activation on ECs [32] has been shown to maintain the expression of enzymes responsible for the synthesis of PNAd and other scaffolding proteins required for supporting lymphocyte trafficking [34]. There is therefore a clear rationale for targeting the LTβR signalling pathway to induce TA-HEVs in tumours for therapeutic benefit. Early work by Lukashev *et al*. examined the impact of LTβR agonist treatment on *in-vivo* tumour growth using an agonistic anti-LTβR mAb (CBE11) [33]. Using xenograft models of colorectal carcinoma CBE11 was found to reduce the growth of 2/6 independent orthotopic xenografts [33]. CBE11 was also found to enhance lymphocyte infiltration in mice bearing syngeneic CT26 tumours [33]. However, the impact on CT26 tumour growth and whether TA-HEVs form as a consequence of CBE11 treatment was not assessed [33]. CBE11 was also found to inhibit the growth of several tumour cell lines *in vitro* suggesting that the anti-tumour mediated effects may not be related to induction of TA-HEVs [33].

More recent studies have assessed the impact of LTβR agonist treatment and its relation to murine tumour growth and TA-HEV formation (these are summarized in Table 1). Using three tumour cell line models Allen *et al*. showed that LTβR agonist treatment *in vitro* had no impact on tumour cell proliferation or death [35]. This allowed for the de-coupling of the direct anti-tumour effects of LTβR agonist from TA-HEV induction. Subsequent *in-vivo* experiments revealed that LTβR agonist significantly increased the number of TA-HEVs per tumour area in RT2-PNET tumours but not in MMTV-PyMT tumours [35]. Interestingly, combinatory anti-angiogenic and PD-L1 blockade therapy led to the induction of TA-HEVs in both tumour models [35]. The addition of LTβR agonist to this combinatory therapy led to a further significant increase in TA-HEVs in both tumour models [35]. The number of CD8^+^ T cells around tumour vessels was also highest in this triple therapy group. This was accompanied by an increase in apoptosing cells and necrotic areas which was further enhanced by addition of the LTβR agonist [35]. Overall, this suggests that TA-HEV induction in the absence of vessel normalization (via anti-angiogenic therapy) and immune activation (via checkpoint blockade) may drive formation of TA-HEVs (PyMT model) but is insufficient in driving an effective anti-tumour response. Indeed, in glioblastoma (GBM), administration of LTβR agonist alone had no impact on the tumour growth and burden [35]. Only when combined with anti-angiogenic/anti-PD-L1 antibody therapy did this lead to the induction of TA-HEVs and a reduced tumour burden [35]. More recently, Asrir *et al*. examined the impact of LTβR agonist treatment on the extent and quality of the T-cell infiltrate in the MCAprog tumour model [12]. LTβR agonist treatment increased the frequency and numbers of TA-HECs in both WT and Rag2^−/−^ mice suggesting the direct activation of LTβR on tumour endothelial cells irrespective of the presence of immune cells [12]. Interestingly LTβR agonist treatment did not lead to an increase in the number or percentage of CD8^+^ T cells [12]. Instead, a reduction in the frequency of PD-1^high^CD8^+^ exhausted T cells and terminally exhausted CD8^+^ T cells and an increase in the proportion of stem-like CD8^+^ T cells was seen. This suggests that LTβR agonist treatment leads to an alteration in the recruiting functionality of TA-HEVs favouring the recruitment of stem-like T cells. Whilst a significant reduction in tumour weight was reported, MCAprog tumour regression was not reported [12]. Importantly, only when LTβR agonist treatment was combined with dual ICB were there significant increases in CD8^+^ tumour infiltrating lymphocytes (TILs) which were accompanied by improved responses to therapy and tumour regression [12]. The presence of stem-like T cells following LTβR treatment may explain why combined therapy with ICB is more effective as the presence of these cells have been implicated in favourable responses to ICB therapies [36, 37]. Indeed, stem-like T cells have been shown to provide the proliferative burst in response to ICB [36] and further correlate with the efficacy of certain tumour vaccines [38, 39]. The presence of stem-like T-cells surrounding TA-HEVs was also reported in PyMT-bearing mice treated with combined LTβR -agonist, ICB and anti-angiogenic therapy [13]. HEV^+^ human tumour sections from six treatment-naive luminal, Her2^+^, and triple-negative breast cancer patients were also shown to have stem-like T cells surrounding TA-HEVs [13].

**Table 1:** Treatment outcomes of HEV inducing agents in different murine tumour models.

Tumour model	Treatment	Outcome
Ag104Ld-EGFR [40]	Anti-EGFR-hmLIGHT fusion protein +/− PD-L1 blockade	PD-L1 blockade or LIGHT treatment alone failed to control tumours. Additional PD-L1 blockade following LIGHT completely eradicated tumours
MCAprog cell line inoculation [12]	LTβR agonist +/− CTLA4 + PD-1 blockade	LTβR alone reduced tumour weight, no change in percentage and number of CD8^+^ T cells. Increase in proportion of stem-like CD8^+^ T cells and the ratio of stem-like to terminally exhausted CD8^+^ T cells (SLAMF6^+^/TIM3^+^) Combined therapy resulted in significant increases in CD8^+^ TILs which were accompanied by improved responses to therapy and tumour regression
B78-D14 melanoma cell line inoculation [41]	Soluble lymphotoxin alpha (sLTα) or ch14.18-LTa fusion protein	sLTa no impact on tumour growth or T cell infiltrate ch14.18-LTa fusion protein drives tumour regression and increase in T cell infiltrate
PyMT (polyoma middle T oncoprotein) breast cancer, RT2-PNET (Rip1-Tag2 pancreatic neuroendocrine tumours) and glioblastoma (GBM) [35]	LTβR agonist +/− 2 weeks of anti- angiogenic/PD-L1 blockade therapy	LTβR agonist alone no change in TA-HEV tumour area in MMTv-PyMT and no impact on tumour cell death and proliferation Neither LTβR activation alone nor anti- angiogenic/PD-L1 blockade therapy affected tumour growth, but the combination of both treatment modalities reduced tumour burden by more than 60%
B16 Metastasis model [42]	A fusion compound of the cytokine LIGHT and a vascular targeting peptide (LIGHT-VTP) +/− PD-1 blockade	Few or absent HEVs in LIGHT-VTP-treated or untreated mice, respectively (NS) Intralesional MECA79^+^ HEV frequency accompanied by extensive immune cell infiltration was 6-fold higher in combination treatment groups when compared to LIGHT-VTP monotherapy
GFP+ NFpp10-GBM cells [43]	LIGHT-VTP +/− VEGF + PD1 blockade	LIGHT-VTP alone induced TA-HEVs (20% of tumour) and significantly more CD3^+^ immune cells infiltration in tumours. Percentage of CD8^+^ T cells per tumour surface area and extent of GrzB expression was comparable to that of untreated tumours Combined treatment significant increase in CD8^+^ T cells and an upregulation of GrzB expression. Significantly reduced tumour burden as compared to LIGHT-VTP treatment alone
RIP1-Tag5 mice bearing neuroendocrine pancreatic tumours which express SV-40 neoantigen [44]	Unconjugated LIGHT or LIGHT-VTP +/− CTLA4 + PD1 blockade	Unconjugated LIGHT, did not confer a survival advantage relative to the survival of untreated mice LIGHT-VTP treatment led to weight loss (ns) and 4-week survival advantage. Following 7 weeks of treatment, tumours in RIP1-Tag5 mice treated with LIGHT-VTP were similar in appearance to those in untreated mice. More cell death in treatment group LIGHT-VTP plus checkpoint blockade of both CTLA-4 and PD-1 increased the mean survival of mice from 30 weeks (with LIGHT-VTP monotherapy) to 36 weeks
Ag104 fibrosarcoma46 expressing murine H-2Ld (AG104-Ld) [45]	Forced expression of LIGHT on Ag104Ld tumour cell line by retroviral transduction using the pMFG vector or injection of LIGHT cells post transplantation of Ag104Ld cell line	Subcutaneous inoculation with bulk Ag104Ld-LIGHT cells, or with the H10 subclone of Ag104Ld-LIGHT, led to tumour rejection in C3B6F1 mice at inoculating doses as high as 5 × 106 cells. 500-fold higher dose than the lowest dose at which untransfected Ag104Ld showed 100% outgrowth in these mice. high number of naive CD62L^hi^CD44^lo^ 2C T cells were detected inside Ag104Ld-LIGHT tumours but not in parental Ag104Ld tumours
PyMT and E0771 breast cancer [13]	DPAg (VEGFR-2 + PD-1 + LTβR agonist) therapy for 10–13 days (PyMT) or 8 days (E0771) and stopped the treatment and followed tumour growth for 2 weeks	DPAg therapy impaired tumour growth and led to TA-HEV induction in both tumours. On treatment cessation, tumours relapsed within a week with comparable growth rates to control. TA-HEVs regressed within 1 week and declined within 2 weeks accompanied by diminished lymphocyte aggregates
Methylcholanthrene induced fibrosarcoma model [20]	LTβR +/− Treg depletion	LTβR agonist treated tumours (with or without Treg depletion), developed extensive and dense TA-HEVs which permeated into deeper parts of the tumour as compared to Treg depletion alone. LTβR agonist treated tumours did not have significantly more TILs or better control of tumour growth

It is interesting to note that in the MCAprog model, a maturation of TA-HECs was seen only following triple therapy characterized by the expression of LTβR dependant genes and a plump morphology reminiscent of that seen in SLOs [12]. This suggests that immune activation is necessary for maintaining a mature TA-HEC phenotype capable of recruiting TILs and driving effective anti-tumour immune responses. Indeed, the administration of FTY720 (blocks lymphocyte egress from LNs) led to a significant reduction in the recruitment of tumour-infiltrating CD8^+^ T cells further supporting mature TA-HEVs in actively recruiting T cells from the periphery [12]. Examining underlying transcriptional differences between TA-HECs induced by LTβR agonist treatment alone and in the presence of immune activation (following ICB for example) may shed light on which genes/gene pathways are crucial for generating effective TA-HEVs capable of driving effective anti-tumour immune responses. Whilst spontaneously arising TA-HECs were shown to express LTβR, treatment with LTβR agonist alone did not drive the maturation of TA-HEVs to the same extent as to that seen following immune activation by ICB [12]. Why LTβR treatment drives the preferential recruitment of stem-like T cells could also be better understood by comparing transcriptional differences between LTβR induced TA-HECs and spontaneously arising TA-HECs. Finally, whether mature TA-HECs induced by immune activation and LTβR agonist treatment differ from spontaneously arising TA-HECs in terms of their ability to preferentially recruit naïve, central memory or effector memory T cells and how this further relates in the context of effective anti-tumour immunity, remains to be determined.

The notion that LTβR agonist treatment can drive TA-HEV formation which is not associated with increased TILs or enhanced tumour control was recently demonstrated in the MCA-induced fibosarcoma model [21]. 3-dimensional (3D) light sheet fluorescence microscopy imaging of Treg-depleted regressor fibrosarcoma tumours revealed the formation of TA-HEVs which were largely well separated and localized to the outer margins of the tumour [21]. In contrast, LTβR agonist-treated tumours developed extensive and dense TA-HEVs which permeated into deeper parts of the tumour. However, despite clear increases in the extent of TA-HEVs, LTβR agonist-treated tumours did not have significantly more TILs or better control of tumour growth [21]. Again, these data point to a link between T cell activation and development of ‘useful’ TA-HEV.

### Delivery of LIGHT to the TME

The induction of TA-HEVs which are associated with improved tumour control following combined immune activation (via ICB) and administration of LTβR agonist [12, 13, 35] is closely mirrored in instances of TNF superfamily member 14 (LIGHT) delivery to the tumour microenvironment. In addition to signalling through the LTβR pathway, LIGHT also signals through herpes virus entry mediator (HVEM); an important co-signalling pathway in T cells [40] that has also been implicated in lymph node neogenesis [43]. The development of an antibody-guided LIGHT (anti-EGFR-hmLIGHT fusion protein) was shown to enhance TIL entry into EGFR-expressing Ag104Ld tumours [44]. Whilst this was sufficient to control smaller Ag104ld tumours, the effective control of large tumours required combined ICB (PD-L1 blockade) treatment with LIGHT delivery [44]. Whilst in this work the induction of TA-HEVs was not assessed following LIGHT delivery, several studies have examined this in the context of targeting LIGHT to tumour vessels via vascular targeting peptides (VTP) [42, 46, 47 ]. LIGHT-VTP was first shown to drive vessel normalization in the spontaneously arising pancreatic neuroendocrine tumours in RIP1-Tag5 mice, which express the SV40 large T antigen under the control of the rat insulin promoter [46 ]. Importantly, LIGHT-VTP led to the induction of TA-HEVs containing TLS associated with CD3^+^ clusters which otherwise do not form spontaneously [46 ]. It is interesting to note that HVEM signalling and not LTβR signalling following LIGHT delivery was required for the expression of inflammatory cytokines such as IL-1β by macrophages. Indeed, vessel normalization in RIP1-Tag5 mice was previously linked to TGF-β secretion by tumour-resident CD68^+^ macrophages which induced pericyte contractility and restored vascular integrity [48 ]. Furthermore, isolated peritoneal macrophages from mice bred on the same background (C3HeBFe) and activated with LIGHT-VTP was sufficient to induce TA-HEV containing TLS following intraperitoneal delivery to tumour-bearing mice [46 ]. CD4 T cell depletion prior to LIGHT-activated macrophage delivery led to a significant reduction in TA-HEVs, implicating both cell types in TA-HEV induction [46 ]. Overall, this suggests that LIGHT activation of the HVEM signalling pathway can promote vessel normalization that is otherwise not driven by LTβR targeting alone. While TA-HEV could be induced by LIGHT-VTP therapy the effects on tumour control were moderate with reportedly slower tumour progression which at 7 weeks post treatment was comparable to that of untreated mice [46 ]. However, by combining LIGHT-VTP with checkpoint blockade of both CTLA-4 and PD-1 (termed ‘LIGHT-VTP triple therapy’) there was an inhibition of tumour growth, necrotic haemorrhaging and prolonged survival [46 ].

This synergism between LIGHT delivery, vessel normalization and immune activation via ICB (PD-L1 blockade) was further demonstrated in a mouse model of GBM [42]. As with pancreatic neuroendocrine tumours [46 ], LIGHT-VTP therapy led to the normalization of angiogenic tumour vessels in the NFpp10-GBM mouse model, which was characterized by the maturation of pericytes, re-establishment of vessel integrity through VE-Cadherin upregulation and improved tumour perfusion [42]. Importantly, this led to the induction of PNAd ^+^ vessels (which otherwise do not form spontaneously) and significantly more CD3^+^ immune cells per tumour surface area [42]. However, the percentage of CD8^+^ T cells per tumour surface area and extent of granzyme B (GrzB) expression was comparable to that of untreated tumours [42]. Only when combined with VEGF and PD-L1 blockade was there a significant increase in CD8^+^ T cells and an upregulation of GrzB expression [42]. Importantly, triple therapy led to a significantly reduced tumour burden as compared to LIGHT-VTP treatment alone [42]. While LIGHT-VTP therapy alone is largely ineffective at reducing tumour growth [42, 46 ], the normalization of blood vessels following LIGHT-VTP therapy in Lewis lung carcinoma tumour-bearing mice was shown to suppress macro- and microscopic lung colonization following either neoadjuvant or postsurgical LIGHT-VTP treatment [47 ]. LIGHT-VTP therapy was also effective at normalizing pathological blood vessels in the pre-metastatic niche [47 ]. Interestingly in established B16 melanoma lung metastases, LIGHT-VTP monotherapy significantly increased the percentage of CD8^+^/GrzB^+^ effector T cells and reduced the metastatic burden significantly [47 ]. However, TA-HEVs were largely absent following LIGHT-VTP monotherapy in B16 lung metastases suggesting anti-tumour control was not dependent on TA-HEV induction [47 ]. Consistent with the aforementioned studies, combined LIGHT-VTP therapy with ICB led to an increased TA-HEV frequency, extensive immune cell infiltration (>6-fold increase) and a significantly reduced metastatic burden as compared to LIGHT-VTP monotherapy alone [47 ].

Taken together it is clear that LTβR agonist or LIGHT monotherapy whilst sufficient in inducing TA-HEVs (in some mouse tumour models) is largely insufficient in driving effective anti-tumour immune responses. Furthermore, the maturation of TA-HEVs in response to immune activation following ICB in the MCAprog model [12], supports the notion of a positive feedback loop whereby factors released by activated immune cells drive the formation of TA-HEVs that are functionally capable of recruiting TILs from the periphery. This is similar to the positive feedback loop described in the MCA-induced fibrosarcoma model, whereby Treg depletion drives immune activation leading to the induction of TA-HEVs and an influx of T cells which release factors that promote TA-HEV formation [18, 19]. An important consideration for TA-HEV-inducing therapies is whether the induction of TA-HEVs in the presence of immune activation is self-sufficient once the positive feedback loop is initiated. Using the R26R-Confetti tracer mouse model which can be employed to randomly label and track the fate of individual cells, Hua *et al*. first demonstrated that the majority of arising TA-HEVs do not come from clonally expanding ECs but rather via post capillary venule (PCV) metaplasia [13]. This argues against the presence of a progenitor/specific EC subtype which has been previously described in the LN setting [41, 49 ]. Importantly, by tracking the fate of labelled ECs following the cessation of CTLA4 blockade + LTβR agonist treatment, TA-HEVs were found to promptly transition back to a PCV state concomitant with a significant diminishment in CD3^+^ aggregates and tumour relapse [13]. This suggests that continual treatment is required to maintain TA-HEVs capable of driving effective anti-tumour immune responses. Whether continued LTβR treatment in the absence of immune activation following treatment cessation of CTLA4 blockade + LTβR agonist is sufficient to maintain TA-HEVs and their lymphocyte aggregates which drive control of tumour growth was not determined in this work.

How might immune activation promote the formation of TA-HEVs? Several factors released by immune cells have previously been shown to induce TA-HEVs. In the PyMT tumour model, LTα_1_β_2_ lymphotoxin production by CD8 T cells and NK cells were shown to drive TA-HEV formation via signalling through the LTβR/noncanonical NFkβ axis [13]. In contrast, in B16-OVA tumour-bearing mice the release of homotrimeric LT_α_3 by effector lymphocytes and signalling through TNFRs was shown to drive PNAd expression [14]. In Treg-depleted MCA-induced fibrosarcomas, the release of TNF by T cells and activation of TNFRs was shown to be necessary for TA-HEV formation in tumours [18]. Interestingly, in both tumour models blockade of LTβR signalling did not abrogate PNAd expression or the extent of immune infiltration [14, 18]. Overall, this suggests that LTβR signalling which is required for maintaining functional HEVs in SLOs [34] is dispensable in some tumour models.

## Conclusion

**Figure 2:**
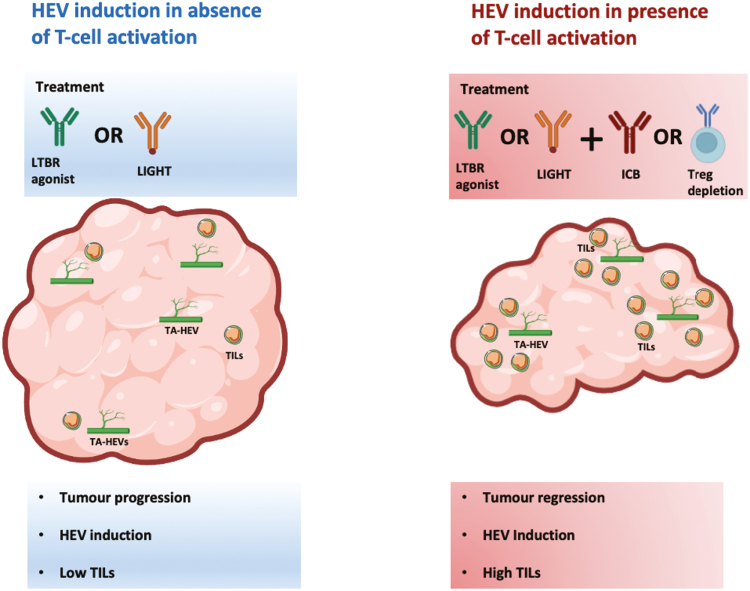
HEV induction in absence vs presence of T-cell activation. In the absence of T-cell activation, LTβR agonist or LIGHT have been shown to effectively induce TA-HEVs in several pre-clinical mouse models. However, this does not lead to improved control of tumour growth or enhanced infiltration of tumour infiltrating lymphocytes (TILs). Only when coupled with immune activation via immune checkpoint blockade (ICB) or Treg depletion does this lead to enhanced control of tumour growth and an increase in TILs.

Recent efforts have shed light on how TA-HEVs arise [13] and the underlying transcriptional similarities and differences between LN-HECs and tumour HECs [12, 13]. Importantly the functional immune recruiting capabilities of TA-HEVs have been directly observed and characterized [12]. Future efforts should focus on understanding which cancers this immunotherapeutic approach would benefit and for how long such treatments should be administered. It is clear that TA-HEV induction in the absence of immune activation is likely to be insufficient in driving lymphocyte infiltration and successful control of tumour growth. Less immunogenic tumours with poor immune cell infiltrates such as GBM may require additional vessel normalization (via anti-angiogenic therapy) together with TA-HEV induction and immune activation as demonstrated in GBM mouse models [35, 42]. Nevertheless, TA-HEV induction in the absence of immune activation is unlikely to be effective in turning cold tumours into hot tumours without concomitant immune cell activation (Figure 2). The extensive TA-HEV networks that form in MCA-induced fibrosarcomas treated with LTβR agonist alone in the absence of Treg depletion and the lack of tumour control thereof are a clear testament to this notion [21].

## Data Availability

Not applicable.

## Abbreviations:

3D: 3-dimensional
CD62L: L-selectin
CRC: colorectal cancer
EGFR: epidermal growth factor receptor
GBM: glioblastoma
Grzb: granzyme B
HEVs: high endothelial venules
HVEM: herpes virus entry mediator
i.p.: intraperitoneal
ICB: immune checkpoint blockade; iLN
HECs: inflamed LN HECs
LIGHT: tumour necrosis factor superfamily member 14
LLC: Lewis lung carcinoma
LN: lymph node
LTβR: lymphotoxin β receptor
Lymphotoxin alpha: LTα
MCA: methylcholanthrene
MSI: microsatellite instability
NSCLC: non-small cell lung cancer
OVA: ovalbumin
PCV: post capillary venule
PNAd: peripheral node addressin
s.c.: subcutaneous
scRNA-seq: singe-cell RNA sequencing
SLOs: secondary lymphoid organs
SV40 Tag: large-T antigen of simian virus 40
TA-HECs: tumour-associated high endothelial cells
TA-HEV: tumour associated HEV
TIL: tumour infiltrating lymphocyte
TLS: tertiary lymphoid structures
TNF: tumour necrosis factor
TNFR: tumour necrosis factor receptor
Tregs: regulatory T cells
VEGF: Vascular endothelial growth factor
VTP: vascular targeting peptides
